# Cellulose Nanofibers Enhanced the Physicochemical Properties of Tannin Fe^3+^ Chitosan Composite Films for Tomato Preservation

**DOI:** 10.3390/gels12040333

**Published:** 2026-04-16

**Authors:** Panpan Feng, Jianguo Lin, Yan Ran, Yingying Zhang, Jiaxin Xu, Yuxin Cheng, Yuanyuan Liu

**Affiliations:** Guizhou Key Laboratory of New Quality Processing and Storage of Ecological Specialty Food, College of Liquor and Food Engineering, Guizhou University, Guiyang 550025, China; e4875fengpp@163.com (P.F.); 15057786269@163.com (J.L.); 15213622020@163.com (Y.R.); 18745505903@163.com (Y.Z.); 15775298498@163.com (J.X.)

**Keywords:** cellulose nanofibers, metal-phenolic nano, coating, performance improvement, fruit preservation

## Abstract

To address inherent limitations of chitosan-based edible films, including inadequate mechanical strength and poor moisture resistance, cellulose nanofibers (CNF) were employed as a synergistic film-forming component to partially substitute chitosan in the fabrication of ternary composite films (denoted as CSTF-CNFs). This approach was based on a previously developed chitosan matrix modified with tannin-Fe^3+^ nanoparticles (TF). It was hypothesized that CNF could function as a reinforcing scaffold to improve the dispersion of TF within the film matrix and, through hydrogen bonding and physical entanglement, form an interpenetrating fiber network with chitosan, thereby enhancing the structural and barrier properties of the films. The present study systematically evaluated the influence of varying CNF substitution ratios (0–30%) on the physicochemical characteristics of the resulting composite films and their performance in tomato preservation. The results demonstrated that an appropriate CNF incorporation facilitated the formation of a dense, cross-linked network with chitosan and TF via hydrogen bond interactions, significantly improving both mechanical strength and water resistance. Among all formulations, the CSTF-CNF20 film exhibited optimal comprehensive performance, achieving the highest tensile strength of 27.60 MPa. Moreover, its swelling ratio markedly decreased from 675.5% (CSTF-CNF0) to 120.9%, while the water contact angle increased to 113.7°, and the DPPH radical scavenging activity remained above 85%. Tomato preservation assays revealed that, in comparison with the untreated control and polyethylene film-wrapped groups, the application of CSTF-CNF20 coating effectively mitigated the decline in weight loss and firmness, preserved surface color integrity, and resulted in the highest L* value alongside the lowest soluble solids content. These findings suggest that the synergistic integration of CNF with nano-scale metal–phenolic networks offers a viable strategy for developing high-performance chitosan-based edible films. The CSTF-CNF20 composite film holds significant promise for application in the postharvest preservation of fruits and vegetables.

## 1. Introduction

Fresh fruits and vegetables are susceptible to browning reactions, texture softening, and spoilage during postharvest storage and transportation due to respiration, water loss, and microbial contamination [1]. Consequently, extending shelf life and maintaining sensory quality while ensuring safety has become a core issue in fruit and vegetable preservation research. Unlike traditional petroleum-based plastic food packaging, which is difficult to degrade [2], coating preservation, as a gentle technology that forms a semi-permeable membrane on the surface of fruits and vegetables, offers advantages in regulating gas and moisture flux, reducing oxidation and microbial contamination [3,4], and minimizing respiration rates and ripening-related metabolism [5], while also being environmentally friendly, edible, and degradable [2]. Therefore, coating preservation exhibits comprehensive advantages of simplicity, edibility, and consumer-friendliness in postharvest preservation. Compared to single cold chain or chemical treatments, coating technology is more easily integrated into existing processes to achieve preservation functions, making it a key direction for improving the quality maintenance and shelf life of fruits and vegetables [6,7].

Tomatoes are highly favored for their rich vitamin content and sweet-and-sour taste [8]. However, as typical climacteric fruits, tomatoes exhibit vigorous postharvest respiratory metabolism, making them highly susceptible to spoilage. Due to respiratory metabolism, high moisture content, and thin epidermis, the process of storage and transportation leads to substantial water loss, contributing to postharvest losses exceeding 30% [9]. Developing safe and efficient coating materials suitable for tomato preservation is therefore of great significance for reducing postharvest losses and maintaining the commercial value of the fruit. In recent years, chitosan-based coatings have shown promising potential for tomato preservation. However, due to the slow diffusion rate of chitosan molecules within the film, chitosan films alone often fail to achieve satisfactory results in practical applications, necessitating the introduction of auxiliary components to overcome this limitation [10].

Chitosan, a naturally occurring alkaline polysaccharide, possesses excellent film-forming properties and broad-spectrum antimicrobial activity. It stands out among various edible coating materials and has been extensively studied for the surface coating preservation of fruits such as apples, citrus, and mangoes [11,12,13]. Recent progress indicates that compositing with metal complexes or plant extracts can synergistically enhance antimicrobial, antioxidant, and structural properties while maintaining safety [14]. However, films composed solely of chitosan suffer from drawbacks such as low mechanical strength and poor water resistance, limiting their application [15]. Furthermore, under high relative humidity, its hydrophilicity and the plasticizing effect of bound water lead to network relaxation and increased water vapor permeability [16], further restricting the long-term effectiveness of chitosan in high-humidity scenarios like cold chains. To overcome these shortcomings, nanomaterial modification has become an important approach: for instance, montmorillonite, graphene oxide, and metal nanoparticles can increase film density and interfacial energy, significantly improving mechanical and barrier properties [17,18,19,20]. In our previous research, composite materials were prepared by combining tannic acid with ferric iron (Fe^3+^). The abundant phenolic hydroxyl groups in these composites enabled them to act as crosslinking agents, which, via hydrogen bonding, restructured the polymer network and led to substantial improvements in the barrier properties, antioxidant activity, mechanical performance, and antimicrobial effects of chitosan-based composite films [21]. It should be noted that relying solely on exogenous nanofillers may still be limited by dispersion and compatibility issues; excessively high filler loads can also lead to drawbacks such as weakened mechanical strength and reduced transparency of the film [22]. Therefore, optimizing the composition of the film-forming polymer system to promote the uniform dispersion of nanomaterials while achieving simultaneous improvement in mechanical properties, barrier performance, and moisture resistance is crucial for the practical application of chitosan films.

Derived from renewable natural resources such as plant biomass, bacteria, and algae, cellulose nanofibers (CNF) possess a high specific surface area, facile surface modifiability, excellent mechanical strength, and good biosafety. These properties have positioned them as key components in active food packaging formulations for green packaging and edible films [23,24]. Thanks to the abundant hydroxyl groups on their surface, CNFs are highly hydrophilic and reactive. They readily form dense network structures with film-forming matrices like chitosan, sodium alginate, and gelatin through hydrogen bonding, electrostatic interactions, or chemical crosslinking. Consequently, the tensile strength, Young’s modulus, and thermal stability of the composite films are significantly improved [25], leading to a synergistic improvement in mechanical and barrier performance [26,27]. In recent years, the application of CNF in active packaging has expanded. Its three-dimensional network structure can serve as a carrier for functional components such as metal nanoparticles, polyphenolic compounds, and essential oils, endowing composite films with active functions like antioxidant, antimicrobial, and gas regulation properties through controlled release behavior [28,29,30]. However, excessive CNF addition can easily lead to agglomeration and phase separation; therefore, optimizing the compatibility of CNF within the film system is crucial. Based on the above issues, cellulose nanofibers (CNF) hold promise as an ideal material to address the dispersion challenges of TF due to their unique structural characteristics. Introducing CNF into the chitosan-tannin iron system is expected to simultaneously improve the dispersion of nanofillers and the network structure of the film-forming matrix through intermolecular interactions, providing a new approach to overcome the performance limitations of existing chitosan-based composite films.

Therefore, this study aims to construct a ternary chitosan-tannin-Fe^3+^-CNF composite film system by employing CNF as a synergistic film-forming agent to partially substitute chitosan. The key questions to be addressed are whether CNF can act as a carrier to promote the dispersion of TF within the film system through molecular interactions, and whether it can construct an interpenetrating fiber network structure with chitosan molecules via hydrogen bonding and physical entanglement, thus enhancing the overall performance of the films. To this end, we systematically investigated the effects of varying CNF substitution ratios on the microstructure, mechanical properties, barrier characteristics, water resistance, and functional activities of the chitosan–tannin–Fe^3+^ composite films. Furthermore, the practical application performance of these films was evaluated through postharvest tomato preservation experiments. Through this work, we aim to explore the potential of this ternary system and contribute insights toward the development of active food packaging film materials.

## 2. Results and Discussion

### 2.1. SEM Analysis of CSTF-CNFs Composite Films

**Figure 1:**
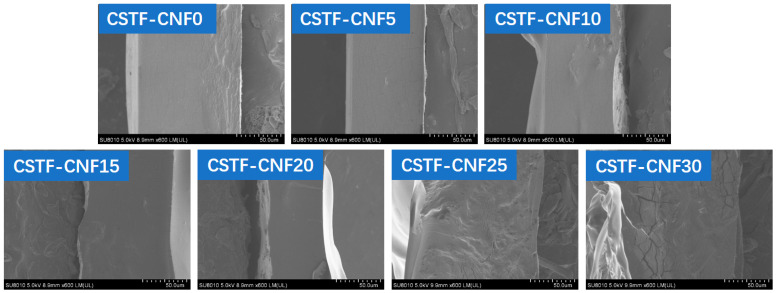
SEM images of CSTF-CNFs composite films with different CNF contents.

SEM imaging revealed that varying the CNF substitution ratio substantially altered the microstructure of the composite films (Figure 1). At a substitution ratio of 0%, the CSTF-CNF0 film showed a relatively smooth and regular cross-section. When the CNF ratio was increased to 5–10%, the CSTF-CNF5 and CSTF-CNF10 films began to exhibit slightly increased roughness. In the CSTF-CNF15 and CSTF-CNF20 films, a decrease in internal smoothness was observed; however, the structure remained highly dense, with observable micro-aggregated skeleton-like textures, indicating favorable hydrogen bonding interactions between CNF and the chitosan matrix at this stage. However, a CNF substitution ratio of 25% led to a significant increase in cross-sectional roughness and the formation of extensive regional aggregates. When the substitution ratio was further increased to 30%, the film structure underwent obvious phase separation, exhibiting a highly irregular cross-sectional morphology with distinct cracks, uneven thickness, and significantly increased roughness. This was attributed to the poor dispersion of excess CNF within the chitosan matrix, leading to fiber agglomeration and disruption of the polymer matrix continuity and compatibility [31]. The results indicated that the incorporation of an appropriate amount of CNF (15–20%) could optimize the microstructure of the composite films, whereas excessive addition (≥25%) compromised the structural integrity of the films.

### 2.2. FTIR Analysis of CSTF-CNFs Composite Films

**Figure 2:**
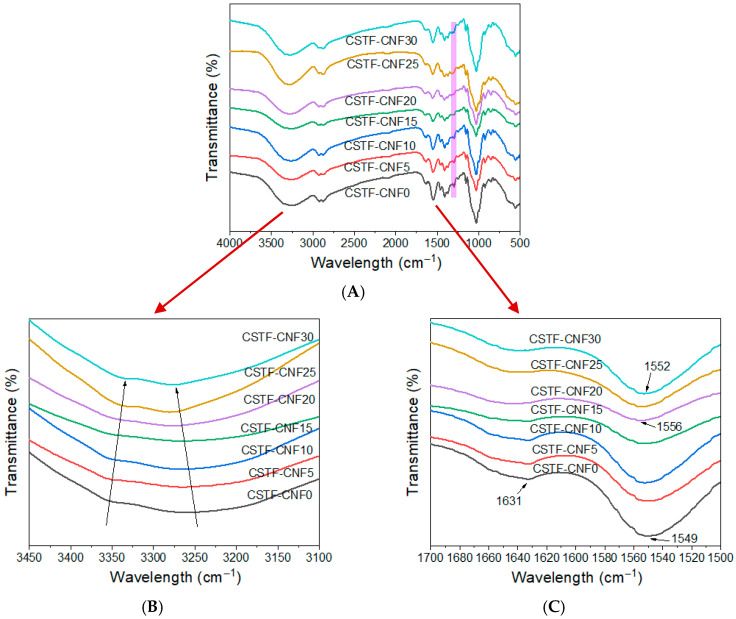
FTIR spectra of CSTF-CNFs composite films with different CNF contents in the ranges of (**A**) 500–4000 cm^−1^, (**B**) 3450–3100 cm^−1^, and (**C**) 1700–1500 cm^−1^.

FTIR spectroscopy offers a reliable approach for analyzing both the interactions between polymers and nanomaterials and the resulting surface chemical changes. The results are shown in Figure 2A. The film without CNF (CSTF-CNF0) exhibited multiple characteristic absorption peaks. The absorption peak in the 950–1100 cm^−1^ range can be ascribed to the β-glycosidic linkages of chitosan [32]. As a cationic polysaccharide, chitosan showed characteristic absorption peaks attributable to its amino groups: the amide I band at 1630 cm^−1^ (C=O stretching and N-H deformation) and the amide II band at 1550 cm^−1^ (N-H deformation and C-N stretching) [33,34]. With the gradual increase in CNF substitution, the peak intensity at 1298 cm^−1^, caused by the C-N vibration of amino groups, gradually weakened. In all samples, a broad and intense absorption band appeared in the 3100–3400 cm^−1^ region, which is attributed to the combined stretching vibrations of O–H (hydroxyl) and N–H (amino) groups [35]. The magnified view in Figure 2B shows that CSTF-CNF0 had absorptions at 3350 and 3250 cm^−1^. Upon CNF incorporation, these peaks underwent red-shift and blue-shift, respectively, with CSTF-CNF5 showing the most significant blue-shift at 3260 cm^−1^. In the 1500–1700 cm^−1^ region (Figure 2C), the addition of CNF led to an initial blue shift in the amide II peak from 1549 cm^−1^ to 1556 cm^−1^ (CSTF-CNF20), followed by a further shift to 1552 cm^−1^. Concurrently, the amide I band at 1631 cm^−1^ showed a gradual decline in intensity. The aforementioned peak shifts and intensity variations indicate that the introduction of CNF altered the chemical environment surrounding the hydroxyl, amino, and amide groups within the films. Combined with existing studies, intermolecular hydrogen bonds can form between chitosan and CNF through hydroxyl and amino groups, while tannic acid can further establish a multi-hydrogen-bonding network with chitosan and cellulose. Additionally, coordination interactions can occur between Fe^3+^ and tannic acid [36,37,38]. Therefore, the CSTF-CNF series films exhibit enhanced intermolecular interactions involving CNF, the tannin-iron complex, and chitosan, with the CSTF-CNF5 film demonstrating the strongest interactions.

### 2.3. Mechanical Properties of CSTF-CNFs Composite Films

**Figure 3:**
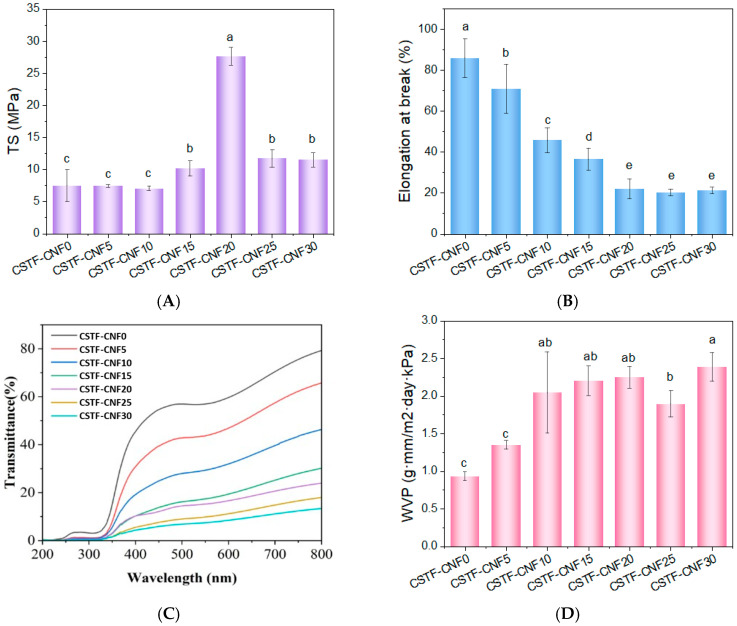
(**A**) Tensile strength, (**B**) elongation at break, (**C**) light transmittance, and (**D**) water vapor permeability of CSTF-CNFs composite films with different CNF contents. Different lowercase letters indicate significant differences among groups (*p* < 0.05, Duncan’s multiple range test).

Excellent mechanical properties are fundamental for composite films to exert their preservation effects during application. Figure 3A,B present the mechanical properties of chitosan–tannin–iron composite films as a function of CNF substitution. The CNF-free film (CSTF-CNF0) showed a tensile strength of 7.48 MPa and an elongation at break of 77.30%, reflecting a typical balance between brittleness and ductility. Within the range of CNF content increasing from 5% to 10%, the tensile strength of the films did not change significantly compared to CSTF-CNF0, but the elongation at break significantly decreased to 45.65%. When the substitution ratio reached 15–20%, the mechanical properties of the composite films underwent a significant transformation. The tensile strength sharply increased from 10.12 MPa (CSTF-CNF15) to 27.60 MPa (CSTF-CNF20), representing an approximately 269% increase compared to CSTF-CNF0, while the elongation at break decreased to 21.86%. For the CNF-free system, the crosslinked network is predominantly constructed via hydrogen bonding and coordination interactions involving chitosan molecular chains and tannin-iron nanoparticles [39]. Upon the introduction of CNF, the nanofibers, with their high aspect ratio and abundant surface hydroxyl groups, not only form hydrogen bonds and physical entanglements with chitosan molecular chains but also interact with tannin-iron nanoparticles [40], thereby constructing a three-dimensional network in which CNF interpenetrates the chitosan-tannin-iron matrix. This interpenetrating network effectively increases the crosslinking density of the system, restricts the free movement of polymer molecular chains, and achieves mechanical reinforcement through intermolecular stress transfer mechanisms [41,42]. This result is consistent with the molecular aggregation morphology observed by SEM and the strong hydrogen bonding interactions confirmed by FTIR. This result was consistent with the molecular aggregation morphology observed by SEM and the strong hydrogen bonding interactions confirmed by FTIR.

However, when the CNF substitution ratio exceeded 20%, the tensile strength showed a significant downward trend (11.70 MPa at 25%, 11.47 MPa at 30%), while the elongation at break remained at a relatively low level of 20–21%. This was attributed to fiber agglomeration and exacerbated phase separation caused by excessively high CNF content, forming stress defect points [43], consistent with the increased surface roughness and decreased structural uniformity observed for the 30% sample in SEM. Furthermore, the elongation at break of the composite films showed a negative correlation with CNF content, possibly because CNF, as a rigid network skeleton, restricted the free movement space of chitosan molecules, leading to decreased ductility [44].

### 2.4. Light Transmittance of CSTF-CNFs Composite Films

Figure 3C illustrates the light transmittance of the composite films. In the visible region (400–800 nm), CSTF-CNF0 displayed excellent transmittance, owing to the well-ordered structure formed by its chitosan molecular chains. As the CNF substitution ratio increased, the transmittance showed a significant, gradual decreasing trend, reaching a minimum of approximately 10% at 30% substitution, with the films gradually transitioning from transparent to translucent and even opaque. This was because the incorporation of CNF led to aggregation through molecular interactions with chitosan and tannin-iron, decreasing the uniformity of the internal film structure, and the interfaces of multiphase aggregates caused light scattering, resulting in decreased transmittance [45]. Notably, all samples exhibited extremely low transmittance (<5%) in the UV region (<350 nm), indicating excellent UV-shielding properties of the films, which is of positive significance for preventing photo-oxidative deterioration of food.

### 2.5. Water Vapor Permeability of CSTF-CNFs Composite Films

To assess the influence of CNF substitution on barrier properties, the water vapor permeability (WVP) of the chitosan–tannin–iron composite films was measured, with results presented in Figure 3D. CSTF-CNF0 exhibited the lowest WVP value (0.93 g·mm/m^2^·day·kPa), reflecting outstanding water vapor barrier capability. This behavior is attributed to the dense hydrogen-bonding network established by the abundant hydroxyl and amino groups on the chitosan molecular chains, combined with the low free volume of the film [46]. As the CNF substitution ratio increased to 10%, the WVP showed a significant, gradual upward trend (*p* < 0.05), increasing from 1.35 for CSTF-CNF5 to 2.04 for CSTF-CNF10, followed by a relatively gentle increase. This phenomenon indicated that the introduction of CNF significantly compromised the moisture barrier performance of the films, possibly because the increased CNF content led to higher roughness and porosity within the film, allowing water vapor molecules to permeate more easily through fiber-matrix interfaces and micropores [31].

### 2.6. Water Contact Angle of CSTF-CNFs Composite Films

**Figure 4:**
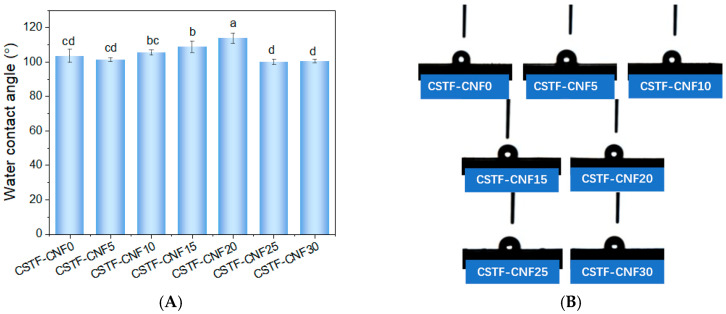

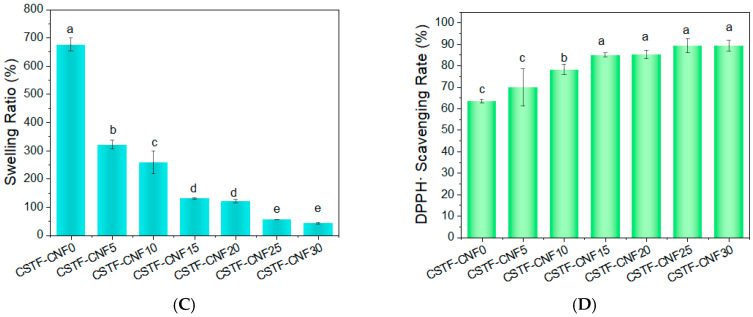
(**A**) Water contact angle values, (**B**) digital photographs, (**C**) swelling ratio, and (**D**) DPPH radical scavenging activity of CSTF-CNFs composite films with different CNF contents. Different lowercase letters indicate significant differences among groups (*p* < 0.05, Duncan’s multiple range test).

As shown in Figure 4A,B, the surface wettability of the CSTF-CNFs composite films varied with CNF content. CSTF-CNF0 exhibited a WCA of approximately 103.5°, indicating a degree of hydrophobicity. This behavior is attributed to the dense arrangement of chitosan molecular chains crosslinked by tannin-iron nanoparticles and the formation of hydrophobic coordination structures. When the CNF substitution ratio was increased, the WCA initially increased before decreasing, with no significant change observed within the 5–10% range.

However, the WCA value of CSTF-CNF15 increased to 108.7°, and it reached a peak of approximately 113.7° for CSTF-CNF20. When the CNF content continued to increase, the WCA value of the films decreased to around 100°. This could be because at low CNF content (5–10%), the fibers were uniformly dispersed in the chitosan matrix, having a limited impact on surface wettability. When the CNF content further increased (10–20%), they interacted and crosslinked with chitosan molecular chains along with tannin-iron, increasing the fibrous interlacing structure within the molecular network and enhancing film roughness [47]. Furthermore, hydrogen bonding between the hydroxyl groups on the CNF surface and chitosan/tannin-iron reduced the exposure of free hydrophilic groups, promoting enhanced surface hydrophobicity [48]. When the CNF content was too high, local aggregation and phase separation occurred due to decreased compatibility, leading to reduced intermolecular interactions and exposure of hydrophilic groups on the surfaces of the two polymer molecules, resulting in the observed decrease in WCA values.

### 2.7. Swelling Properties of CSTF-CNFs Composite Films

The swelling ratio measurements revealed the evolution of the water resistance of the composite films with varying CNF substitution ratios. The results are shown in Figure 4C. CSTF-CNF0 exhibited a high swelling ratio of 675.5%, demonstrating strong hydrophilicity, which was attributed to the strong adsorption of water molecules by the numerous free hydroxyl and amino groups on the chitosan molecular chains [49]. As the CNF substitution ratio increased to 30%, the swelling ratio showed a significant, gradual downward trend, decreasing from 320.7% for CSTF-CNF5 to 120.9% for CSTF-CNF20 and 42.7% for CSTF-CNF30, suggesting that CNF addition significantly improved film water resistance. This is primarily ascribed to the formation of a dense hydrogen bond network via the abundant hydroxyl groups on the CNF surface interacting with chitosan and tannin-iron nanoparticles, which effectively reduced the number of free hydrophilic groups capable of binding water molecules [50]. Additionally, cellulose nanofibers themselves have a low swelling ratio, and partially substituting chitosan with CNF effectively restricted the swelling behavior of the films.

### 2.8. Antioxidant Activity of CSTF-CNFs Composite Films

Figure 4D presents the DPPH radical scavenging activity of the chitosan–tannin–iron composite films at varying CNF substitution ratios. CSTF-CNF0 achieved a scavenging rate of 63.28%, which is largely attributable to the strong radical scavenging capacity of tannic acid nanoparticles coupled with the inherent antioxidant activity of chitosan. The combined effect of these two components results in excellent antioxidant performance of the composite film [51,52]. As the CNF substitution ratio increased, the scavenging rate showed a trend of rapid increase followed by gradual stabilization: CSTF-CNF10 significantly increased to 78.16%. After the CNF substitution ratio reached 15%, the DPPH scavenging rate of the films continued to increase to above 85% and stabilized. The results demonstrated that the incorporation of CNF effectively enhanced the antioxidant performance of the composite films. The main reason could be that the CNF network structure promoted the dispersion of tannin-iron nanoparticles through molecular coordination, allowing them to be uniformly distributed on the fiber surfaces and within network voids, increasing the exposure of active sites and achieving enhanced antioxidant activity [53].

Based on the comprehensive analysis of micromorphology, molecular interactions, and performance, 20% CNF substitution ratio was identified as the optimal formulation for this composite system, achieving the best balance between performance and structural integrity. Therefore, CSTF-CNF20 was selected for subsequent preservation experiments.

### 2.9. Tomato Preservation Experiments

**Figure 5:**
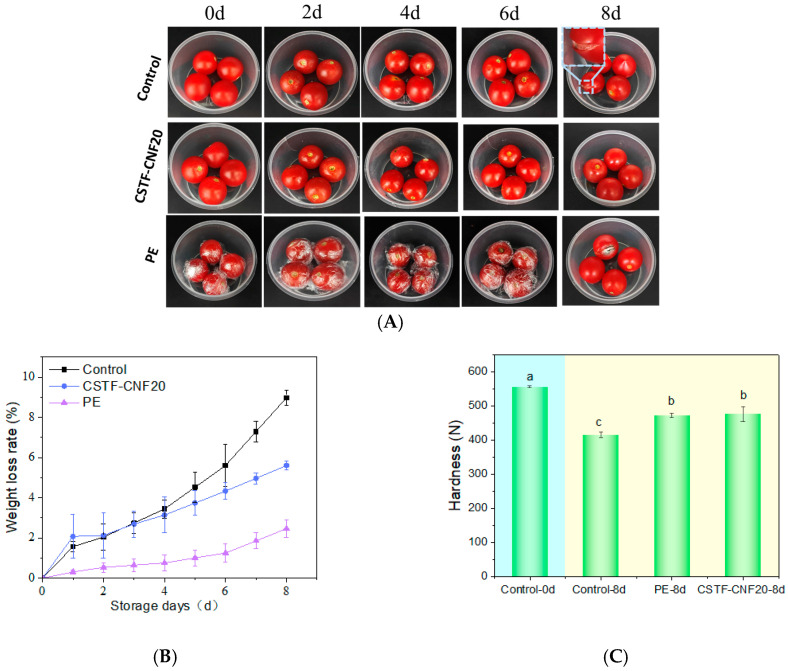
(**A**) Digital photographs, (**B**) weight loss, and (**C**) hardness values of tomatoes in the control group, CSTF-CNF20 coating group, and PE film wrapping group during the preservation experiment. Different lowercase letters indicate significant differences among groups (*p* < 0.05, Duncan’s multiple range test).

In the tomato preservation experiment, the inhibitory effects on postharvest quality deterioration of tomatoes in the control group, coating group (CSTF-CNF20), and PE film wrapping group (PE) were observed through digital photographs. The results are shown in Figure 5A. On day 0, all three groups of tomatoes exhibited an excellent initial state, characterized by plump and rounded shapes, bright red color, and glossy surfaces. As storage time progressed, the degree of quality deterioration varied significantly among the groups. With extended storage time, slight browning was observable at the stem ends of tomatoes in all treatment groups. Notably, by the 8th day of storage, some tomatoes in the control group and PE exhibited obvious fruit rupture and mold growth. The mold growth in the PE might be attributed to the inability to expel moisture generated by respiration, creating a high-humidity environment conducive to mold proliferation [43]. In contrast, the strong antioxidant activity of the tannin-iron nanoparticles in the coating delayed epidermal oxidative browning, and synergistically with the inherent antimicrobial properties of chitosan, inhibited microbial proliferation on the fruit surface [54,55]. The above results demonstrated that the coating treatment possessed a good capacity for maintaining the appearance and morphology of the fruits. Samples that did not undergo spoilage were used for subsequent index measurements.

The weight loss results of tomatoes during storage are shown in Figure 5B. An increasing trend in weight loss rate was observed across all three groups over the storage period, but with significant differences. The control group lost weight most rapidly, reaching 9.0% on day 8, because the absence of physical protection allowed continuous water loss through transpiration and respiration [56,57]. The PE film group showed the slowest weight loss, only 2.5% on day 8, benefiting from the excellent water barrier properties of the PE film. The CSTF-CNF20 coating group was in the middle, reaching 5.6% on day 8, which was 38% lower than the control group but significantly higher than the PE film group. The results indicated that the physical barrier formed by the chitosan-tannin-iron-CNF network could effectively reduce water evaporation but did not completely impede water passage. This favorable water vapor permeability could avoid anaerobic respiration, maintain normal physiological metabolism of the fruit, and, to some extent, benefit tomato preservation.

Fruit firmness is a key determinant of both economic value and sensory quality for fresh tomatoes. The results in Figure 5C reveal that after 8 days of storage, firmness progressively declined across all treatment groups. This deterioration is partly explained by the breakdown of nutrients (e.g., polysaccharides and proteins) and cell wall pectin resulting from respiratory metabolism [58]. On the other hand, water loss led to loss of cell turgor pressure, causing tomato softening, ultimately resulting in a gradual decrease in tomato firmness [59]. Notably, the firmness of both the CSTF-CNF20 coating group and the PE was significantly higher than that of the control group, and there was no significant difference between the two groups. The CSTF-CNF20 group achieved firmness retention comparable to the PE film group under conditions of higher water loss, suggesting that this active film could modulate respiratory metabolic intensity through physiological regulatory mechanisms.

**Figure 6:**
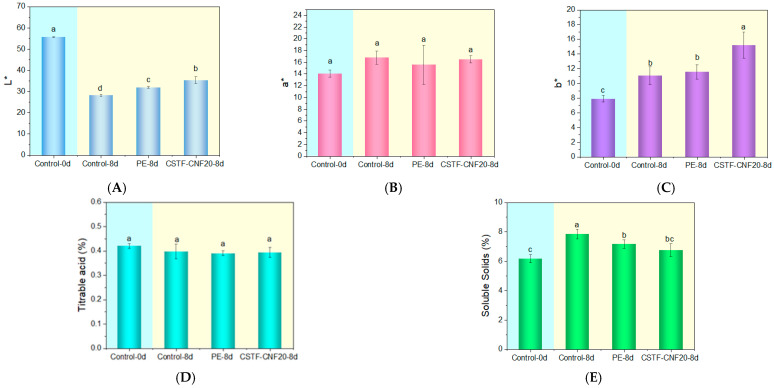
(**A**) L* value, (**B**) a* value, (**C**) b* value, (**D**) titratable acidity content, and (**E**) soluble solids content of tomatoes in the control group, CSTF-CNF20 coating group, and PE film wrapping group during the preservation experiment. Different lowercase letters indicate significant differences among groups (*p* < 0.05, Duncan’s multiple range test).

The external color of fruit decisively influences consumer acceptance. Color differences were evaluated by measuring the lightness (L*), redness (a*), and greenness (b*) of the tomatoes. The results are shown in Figure 6A.

After 8 days of storage, the L* values of all samples decreased to 28 for the control group and 32 for the PE film group. In contrast, the L* value of the CSTF-CNF20 coating group only decreased to 35, with the degree of retention being significantly higher than the other two groups (*p* < 0.05), indicating that this active composite film effectively delayed the loss of surface glossiness in tomatoes. Postharvest color deterioration in tomatoes primarily stems from changes in surface light reflection characteristics due to water loss-induced shrinkage, damage to the epidermal wax layer, and alterations in carotenoid content [57,60,61].

The a* values of all samples increased after storage, with no significant differences among the groups (Figure 6B), demonstrating the enhanced redness of the tomatoes. This might be due to chlorophyll degradation and lycopene accumulation [57,62,63]. The b* values of the control group and PE film group both increased significantly to approximately 11 (Figure 6C), with no significant difference between the two groups (*p* > 0.05). This result indicated that the yellowness of tomatoes increased after storage, possibly originating from carotenoid biosynthesis and accumulation [64]. However, the b* value of the CSTF-CNF20 coating group increased to 15.2, significantly higher than the other two groups (*p* < 0.05), which might be related to the color of tannin-iron within the coating. The above results demonstrated that because the CSTF-CNF20 coating allowed moderate gas exchange, supporting ethylene-mediated normal ripening and lycopene biosynthesis, while antioxidant protection prevented oxidative degradation of red pigments, it exhibited the best ability to maintain tomato color.

The soluble solids content and titratable acidity of tomatoes are important factors in evaluating their eating quality. Figure 6D shows that after 8 days of storage, the titratable acidity of all treated tomato groups showed a numerical decrease with no significant difference (*p* > 0.05), possibly due to respiratory metabolism consuming organic acids as carbon sources for energy [65]. Figure 6E shows that the soluble solids content of all groups increased after storage, attributed to the degradation of macromolecular polysaccharides into soluble sugars during metabolic activities [62]. The soluble solids content was highest in the control group, followed by the PE, while the value for the CSTF-CNF20 coating group was significantly lower than the first two groups. This result demonstrates that, compared to the PE, the CSTF-CNF20 composite film achieved optimized regulation of tomato metabolic activities through antioxidant protection, moderate gas regulation, and water management. The CSTF-CNF20 film demonstrates strong potential for use in fruit preservation. Additionally, its bio-based and biodegradable properties make it well-suited to address the rising need for sustainable packaging materials.

## 3. Conclusions

To address the key issues of low mechanical strength and poor moisture resistance of chitosan-based edible films, this study utilized cellulose nanofibers (CNF) as a co-film-forming agent to partially substitute chitosan, thereby optimizing the previously developed tannin-Fe^3+^ nanoscale modified chitosan film system. A ternary composite film system comprising chitosan, tannin-Fe^3+^, and CNF was successfully established, and the regulatory effects of different CNF substitution ratios on film structure and properties were systematically elucidated. The results demonstrated that an appropriate CNF substitution ratio facilitated the formation of a dense crosslinked network with chitosan and the tannin-iron complex through hydrogen bonding, significantly enhancing the overall application performance of the composite films. Among the formulations tested, the CSTF-CNF20 film exhibited the best comprehensive performance, achieving maximized mechanical properties along with excellent antioxidant activity and UV-shielding performance. Moreover, the incorporation of CNF substantially improved the water resistance and surface hydrophobicity of the composite films, while also enhancing DPPH free radical scavenging activity. Tomato preservation experiments further confirmed that coating treatment with CSTF-CNF20 effectively delayed weight loss and softening, maintained surface gloss, inhibited mold growth, and supported normal tomato ripening through moderate gas exchange. Notably, this treatment outperformed both the control group and the PE film group in terms of firmness retention and color maintenance. Collectively, this study provides a theoretical basis and technical reference for the nanoscale modification of chitosan-based edible films.

## 4. Materials and Methods

### 4.1. Materials

Chitosan (CS, molecular weight 60–80 kDa, degree of deacetylation ≥ 85.00%) was purchased from Sinopharm Chemical Reagent Co., Ltd. (Shanghai, China). Cellulose nanofibers (CNF) were obtained from Beijing Science & Technology Co., Ltd. (Beijing, China). Sodium citrate was purchased from Guangzhou Xilong Technology Co., Ltd. (Guangzhou, China). Tannic acid (CAS No.: 1401-55-4, molecular weight: 1701.20) was obtained from Aladdin Reagent Co., Ltd. (Shanghai, China). Ferric chloride hexahydrate was purchased from Shanghai Rhawn Chemical Technology Co., Ltd. (Shanghai, China). Glycerol was obtained from Jinshan Chemical Reagent Co., Ltd. (Chengdu, China). Propionic acid (purity ≥ 99.5%) was purchased from Shanghai Macklin Biochemical Technology Co., Ltd. (Shanghai, China). 2,2-Diphenyl-1-picrylhydrazyl radical (DPPH·) was obtained from Shanghai Rhawn Chemical Technology Co., Ltd. (Shanghai, China). Fresh tomatoes (variety: Linglong 88) at the full-ripe stage with similar size, color, and maturity were purchased from Shiban Town Fruit Wholesale Market, Huaxi District, Guiyang City, Guizhou Province, China.

### 4.2. Methods

#### 4.2.1. Preparation of Tannin-Iron Nanoparticles

Tannin-iron nanoparticles (TF NPs) were prepared following the procedure described by Zhang et al. [66]. Briefly, FeCl_3_·6H_2_O solution (0.2 mL, 10 mg/mL) and tannin solution (0.8 mL, 10 mg/mL) were mixed in deionized water (20 mL). Pre-dissolved sodium citrate (20 mg) was then introduced, and the mixture was stirred at room temperature for 1 min, resulting in a stable purple solution. The product was collected by centrifugation (10,000 rpm, 5 min) and re-dispersed in deionized water to obtain the final TF NPs solution.

#### 4.2.2. Preparation of CSTF-CNFs Composite Films

The film-forming base was prepared by dissolving chitosan (CS) in 60 mL of 1% (*v*/*v*) propionic acid at 40 °C with continuous stirring until fully dissolved (25 mg/mL). Following this, the TF solution was added, and the volume was adjusted to 100 mL with water, giving final concentrations of 15 mg/mL for CS and 0.06 mg/mL for TF. To prepare the ternary composite films, cellulose nanofibers (CNF) were introduced to partially replace CS by mass, with substitution ratios of 0%, 5%, 10%, 15%, 20%, 25%, and 30%, while maintaining a constant total solid content. Then, 1.5 mL of glycerol was added, and the mixture was stirred thoroughly. The film-forming solutions were cast into molds and dried at 40 °C using the solution casting method, yielding composite films with varying CNF contents. The resulting composite films were designated as CSTF-CNF0, CSTF-CNF5, CSTF-CNF10, CSTF-CNF15, CSTF-CNF20, CSTF-CNF25, and CSTF-CNF30 according to their CNF substitution ratios.

#### 4.2.3. Fourier Transform Infrared Spectroscopy Analysis of CSTF-CNFs Composite Films

Based on the previous method, with slight modifications [67], the composite films were characterized by Fourier transform infrared (FTIR) spectroscopy using a Nexus 470 spectrometer (Nicolet Co. Ltd., Mountain, WI, USA) in attenuated total reflectance (ATR) mode. Spectral data were collected in the wavenumber range of 4000–500 cm^−1^, and each measurement consisted of 64 cumulative scans.

#### 4.2.4. Micromorphology Analysis of CSTF-CNFs Composite Films

To examine the microstructure, the films were first cryo-fractured in liquid nitrogen. The exposed cross-sections were then proceeded with gold sputter-coating and subsequently examined using a scanning electron microscope (SEM, Sigma 500, Carl Zeiss AG, Oberkochen, Germany).

#### 4.2.5. Determination of Mechanical Properties of CSTF-CNFs Composite Films

According to the method of previous researchers, with slight modifications [68,69], the mechanical properties of the films were evaluated at room temperature using a texture analyzer. Prior to testing, the films were cut into rectangular strips measuring 10 mm × 70 mm, and their thickness was determined as the average of five measurements taken at random positions using a micrometer (precision: 0.001 mm). The initial grip distance was set to 40 mm, and the test was performed at a crosshead speed of 0.5 mm/min. Tensile strength (TS) and elongation at break (EAB) were recorded using Texture Pro CT 1.9.35 software. Each formulation was tested in triplicate.

(1) $$TS\left(MPa\right)=\frac{F}{a\times b}$$

(2) $$EAB\left(\%\right)=\frac{L-L_{0}}{L_{0}}\times 100$$

Here, F represents the maximum tensile force (N), a and b represent the thickness and width of the film (mm), respectively. L and L_0_ refer to the length of the film at break (mm) and the length before break (mm), respectively.

#### 4.2.6. Determination of Light Transmittance and Water Vapor Permeability (WVP) of CSTF-CNFs Composite Films

The absorbance of the films was measured in the range of 200–800 nm using a UV-Vis spectrophotometer (UV-2700 Shimadzu, Kyoto, Japan). The light transmittance of the films was calculated from the absorbance values across the entire wavelength range using the formula [66]

T = 10^−A^ × 100 (3)

Here, T represents transmittance (%); A represents the absorbance value of the film.

Adapted from a previous method with minor modifications [70], For water vapor permeability (WVP) measurement, the composite films were cut into 3 cm × 3 cm pieces. The thickness of each piece was determined as the average of measurements taken at five random positions. A glass bottle containing 3 mL of deionized water was sealed with the film specimen and weighed to record the initial weight (M_0_). The bottle was then placed in a desiccator containing anhydrous silica gel. After 12 h of storage, the bottle was weighed again to obtain the final weight (M_t_). The WVP value was calculated using the following formula:

(4) $$WVP=\frac{(M_{0}-M_{t})\times L}{A\times T\times ∆P}$$

M_0_ and M_t_ are in grams (g), L represents the average thickness of the composite film (mm), A represents the area of the glass bottle mouth (m^2^), T represents the duration of the experiment (days), and ΔP represents the saturated water vapor pressure difference between the inside and outside of the glass bottle at 28 °C (3692.5 Pa).

#### 4.2.7. Determination of Water Contact Angle (WCA) of CSTF-CNFs Composite Films

Static water contact angle measurements were conducted on the composite films using a contact angle instrument (SPCAX3, Beijing Hako Testing Instrument Factory, Beijing, China) to characterize their surface wettability. A 10 µL droplet of ultrapure water was gently deposited onto the film surface. Upon contact, the droplet shape was photographed immediately to minimize the influence of receding angle effects. The static contact angle was then determined as the angle formed between the tangent to the liquid-air interface and the film surface at the three-phase contact point.

#### 4.2.8. Determination of Swelling Ratio of CSTF-CNFs Composite Films

Films were cut into 2 cm × 2 cm pieces, weighed, and recorded as m_0_. Subsequently, they were immersed in deionized water. After 30 min, the pieces were removed, surface moisture was gently blotted with filter paper, and they were weighed again using an analytical balance, recorded as m_1_. The swelling ratio of the samples was calculated according to the following formula [71]:

(5) $$Swelling\;ratio\left(\%\right)=\frac{m_{1}-m_{0}}{m_{0}}\times 100$$

#### 4.2.9. Antioxidant Activity of CSTF-CNFs Composite Films

The antioxidant activity of the composite films was evaluated using the DPPH radical scavenging assay, following the method described by Zhang et al. [31]. Briefly, a pre-weighed film sample was immersed in 2 mL of 0.2 mmol/L DPPH ethanol solution. The mixture was incubated in the dark at room temperature for 90 min. Thereafter, the absorbance of the resulting solution was measured at 517 nm (*A*_1_) using a microplate reader, alongside that of the blank DPPH solution (*A*_0_). The DPPH radical scavenging rate was calculated accordingly.

(6) $$DPPH\cdot scavenging\;rate\left(\%\right)=(1-\frac{A_{1}}{A_{0}})\times 100$$

#### 4.2.10. Tomato Preservation

Fresh tomatoes of similar size, color, and maturity were selected and randomly divided into three groups (control group, CSTF-CNF20 coating group, and commercial polyethylene (PE) film-wrapped group), with three replicates per group and four tomatoes per replicate. For the coating group, tomatoes were immersed in the CSTF-CNF20 film-forming solution for 2 min, then removed and allowed to air-dry naturally to ensure uniform coating coverage on the fruit surface. All samples were stored at room temperature (25 ± 2 °C) for 8 days, during which appearance changes and weight loss were recorded daily. Following the method described by Zhang et al. [31], color parameters, firmness, soluble solids content, and titratable acidity were measured on day 0 and day 8.

#### 4.2.11. Data Analysis

All experiments were performed in triplicate at a minimum, and the results were expressed as mean ± standard deviation (SD). Statistical significance (*p* < 0.05) for datasets (≥3 replicates) was assessed using one-way ANOVA in SPSS v26.0, followed by Duncan’s multiple range test. Graphs were plotted using Origin 2024 software.

## Data Availability

The original contributions presented in this study are included in the article. Further inquiries can be directed to the corresponding author.
